# Social Intervention by the Numbers: Evidence Behind the Specific Public Health Guidelines in the COVID-19 Pandemic

**DOI:** 10.1089/pop.2020.0180

**Published:** 2021-06-08

**Authors:** Michael P. Savage, David L. Fischman, Mamas A. Mamas

**Affiliations:** ^1^ Department of Medicine, Thomas Jefferson University, Philadelphia, Pennsylvania, USA.; ^2^ Centre for Prognosis Research and Department of Cardiology, Keele University, Stoke-on-Trent, United Kingdom.

**Keywords:** COVID-19 pandemic, social distancing, nonpharmaceutical interventions, public health guidelines

## Introduction

Coronavirus disease 2019 (COVID-19) has rapidly become a worldwide pandemic ushering in a global health and economic crisis. In the absence of vaccines or definitive drug therapies, current strategies against COVID-19 rely on preventing the transmission of disease through nonpharmaceutical interventions such as social distancing and proper hand hygiene. For practical and perhaps ethical reasons, the clinical efficacy of these public health measures for managing pandemics has not been demonstrated in randomized controlled clinical trials. Accordingly, guideline recommendations for using nonpharmaceutical interventions are based primarily on observational and modeling studies and on expert opinion. A recent position paper from the World Health Organization (WHO) graded the quality of evidence to be low for the efficacy of social distancing interventions for mitigating pandemic influenza.^1^ Nonetheless, observational studies have credited nonpharmaceutical interventions with slowing the spread of COVID-19 in China and on the west coast of the United States.^2–4^ These apparent salutary effects in the COVID-19 pandemic mirror the benefits of similar interventions observed in prior influenza pandemics.^5^ Further confirmation of the importance of these measures has come with the loosening of social distancing practices and subsequent rapid surge of cases in the Sunbelt region of the United States as the country attempted to reopen.

In addition to general measures such as closure of schools and nonessential businesses, public health guidelines for managing COVID-19 include a series of specific, quantitative recommendations that are the focus of this analysis (Table 1).^6–8^ Among these are explicit rules regarding interpersonal distancing (6 feet), limitation of group gatherings (10 people), duration of quarantine for exposed individuals (14 days), and duration of handwashing (20 seconds). These recommendations have received wide interest from the international medical community and general public. The goal of this analysis is to critically evaluate the evidence basis behind these specific, quantitative nonpharmaceutical interventions that have a prominent role in mitigating the current COVID-19 pandemic.

**Table 1.** Specific Recommendations for Nonpharmaceutical Interventions to Mitigate COVID-19

	Interpersonal distance	Quarantine duration	Mass gatherings	Handwashing duration
CDC^6^	6 feet	14 days	Varies by community	≥20 seconds
WHO^7^	1 meter (∼3 feet)	14 days	Case-by-case basis	40 to 60 seconds
ECDC^8^	—	14 days	Discouraged without quantification	20 to 40 seconds
PCG	—	—	Avoid gatherings of >10 people	—
Agreement Among Public Health Organizations	Slight Discordance	Uniform Concordance	No Uniform Recommendation	Moderate Concordance
Strength of Evidence for COVID-19	Weak	Moderate	Weak	Moderate

CDC, Centers for Disease Control and Prevention; ECDC, European Centre for Disease Prevention and Control; PCG, President's Coronavirus Guidelines for America (www.whitehouse.gov); WHO, World Health Organization.

## Interpersonal Distancing with 6 Feet of Separation

There is a lack of unanimity among health organizations as to the recommended distance for interpersonal spacing (Table 1). The US Centers for Disease Control and Prevention (CDC) states, “COVID-19 spreads mainly among people who are in close contact (within about six feet) for a prolonged period.”^9^ Accordingly, the CDC recommends a 6-foot distance between people. The WHO recommends 1 meter, only about half the distance recommended by the CDC. In contrast, a 2-meter distance is recommended in the United Kingdom while other countries recommend 1.5 meters.^10,11^

The primary mode of transmission is thought to be through droplet transmission when an infected person coughs, sneezes, or talks during close contact. Droplets refer to larger expelled particles that typically travel less than 6 feet before falling to the ground. More controversial is the infective potential of smaller viral-containing particles called “aerosols” (<5–10 mm in diameter), which travel further and stay airborne longer. The measles virus and tuberculosis are familiar pathogens known to spread via aerosols. The capacity of coronaviruses to infect by airborne transmission has been an issue of uncertainty.^12^

The origin of the 1-meter distancing rule endorsed by the WHO stems from work dating back to the 19^th^ century by Professor C. Flugge at the University of Breslau. Using exposed plates at various distances to measure how far expiratory droplets travel, bacteria-containing droplets were observed to settle quickly. It was concluded that infection from droplets other than within a few feet of the “infector” was unlikely.^13^ As late as 1996, a CDC guideline on infectious isolation precautions concurred with the 3-foot distance, noting that droplets “do not remain suspended in the air and generally travel only short distances, usually three feet or less.”^14^ With the new millennium came new evidence questioning old dogma regarding airborne transmission of respiratory infections and the 1 meter (3 foot) recommendation. Olsen and colleagues studied more than 100 people who had potential in-flight exposure to the severe acute respiratory syndrome (SARS) coronavirus.^15^ Of the 23 passengers seated in the same row or in 3 rows directly in front of a symptomatic passenger who unknowingly had SARS, 8 (35%) subsequently developed SARS. In contrast, only 10/88 (11%) passengers seated elsewhere became ill (relative risk, 3.1; 95% CI, 1.4 to 6.9). Of the persons who became ill, 90% were seated more than 36 inches away from the index patient, suggesting that airborne transmission of small viral particles was responsible. Current CDC guidelines now recommend 6 feet of separation for transmission avoidance.^6^

Whether 6 feet of distancing is sufficient has come under increased scrutiny. Quantitative air samples of viral particles taken at 1, 3, and 6 feet from influenza patients detect a greater concentration of larger particles at 1 and 3 feet. Samples at 6 feet are predominantly small particles but have sufficient concentration to infect humans.^16^ The potential for viruses to travel more than 6 feet in air may be affected by several factors. Enclosed indoor spaces often have air currents that facilitate farther transport of particles. Current social distancing recommendations pertain to persons who are standing still. They do not take into account potential aerodynamic effects introduced by movement such as brisk walking, running, or cycling, which create a slipstream behind the person carrying exhaled droplets well beyond 6 feet. Recent studies of respiratory emissions have demonstrated that coughing and sneezing result in exhalation speeds of up to 30 meters/second and create clouds of varying sized droplets that travel up to 8 meters.^17^ Therefore, it appears there are many conditions under which airborne transmission could well exceed 6 feet.

The relevance of these observations for COVID-19 is presently uncertain but studies on the biodynamics of aerosols containing SARS-CoV-2 are emerging. The airborne exposure of COVID-19 from hospitalized patients was evaluated in Wuhan.^18^ Air samples were positive for the virus in 35% of samples taken in the intensive care unit and 12.5% of samples taken from general wards. Notably, the transmission distance for COVID-19 was up to 4 meters. SARS-CoV-2 has been shown to be viable in aerosols for up to 3 hours and on surfaces for up to 3 days.^19^ The potential for airborne transmission is likely greatest in enclosed indoor spaces with poor ventilation. These concerns were expressed in a recent commentary supported by more than 200 scientists urging the WHO to update its guidance on the risk of aerosol transmission of COVID-19.^20^

These contemporary data, including reports with COVID-19, suggest the evidence base for recommending 6 feet of interpersonal spacing in the current pandemic is poor. Although it may be argued that 6 feet is practicable, there is no reassurance that infectious risk is negligible at that distance. A recent systematic review of measures to reduce viral transmission concluded that infectious risk was reduced at a 1 meter distance but that longer distances were associated with even greater benefit; it was estimated that relative risk decreased 2-fold for each additional meter of distance.^21^ The spacing length that can be both safe and pragmatic remains unclear. Analogous to the inverse square law for radiation safety, what is clear is that as one moves further away from the source the risk of exposure lessens progressively.

Increasing concerns that COVID-19 may spread by aerosols, which may be transmitted at distances beyond 6 feet, reinforce the role of mask wearing. Masks are especially important in settings where avoidance of close contact is difficult. In the Harvard hospitals, universal masking of both health care workers and patients was associated with a significant reduction in the infection rate among health care workers.^22,23^ Countries in which the populace have widely adopted face mask use have experienced much lower rates of COVID-19 spread.^24^

## Avoiding Gatherings of More Than 10 People

On March 29, 2020, the President's Coronavirus Guidelines for America recommended avoiding social gatherings of more than 10 people. Most health organizations discourage “mass gatherings” during pandemics without stipulating a threshold number. One reason to avoid mass gatherings is the difficulty of maintaining adequate social distance in such situations. To estimate a safe size for an event would require knowing the prevalence of the illness in the target population. For illustration, if the prevalence of infection is 5%, a gathering of 10 people would have a 40% probability that 1 or more of the attendees would be infected. If the disease prevalence were 15% instead, the probability that 1 or more of the attendees would be infected would rise to 80%. Given the lack of widespread testing and the evidence that most people infected with COVID-19 go undiagnosed because of minimal or no symptoms, the prevalence of the disease is unknown. In the absence of these data, selecting a number at which to cap group gatherings appears arbitrary. Although there is no scientifically definable cap number, the important concept is that the potential for transmission will be directly proportional to the size of the crowd. Therefore, from the risk of transmission standpoint, bigger is not better. It is also important to emphasize that other factors beyond the size of the gathering will influence potential spreading, notably the density of the crowd and the length of exposure time.

Evidence to avoid crowding and public events is limited and this accounts for the lack of expert consensus on the effectiveness of this particular social intervention.^25^ There are no prospective or randomized controlled trials on crowd avoidance. The best evidence against mass gathering comes from observational studies.^26^ The most well-known example is the apparent effect of banning public gatherings and closure of public places on reducing the death rate during the influenza pandemic of 1918.^5^

## Quarantine of Exposed Individuals for 14 Days

Quarantine is defined as the imposed separation or restriction of movement of persons who have been exposed to others with the illness and who are not overtly ill, but who may become infectious to others.^1^ Although the evidence base is judged to be weak, quarantine is considered generally effective in reducing the burden and transmission ability of disease and in delaying the peak of the epidemic.^26^ Studies from prior coronavirus outbreaks such as SARS and MERS (Middle East respiratory syndrome) have consistently found a beneficial effect of quarantine, especially when instituted with other social distancing measures.^27–29^ A recent review found a similar benefit of quarantine in COVID-19, although the certainty of the evidence was low because the only available studies were based on simulation modeling.^29^

Major health organizations concordantly recommend a 14-day quarantine period for COVID-19 exposed individuals (Table 1). The duration of quarantine is determined by the specifics of the incubation period from infection exposure to development of symptoms. A pooled analysis of 181 cases reported a median incubation period of 5.1 days with 97.5% developing symptoms within 11.5 days.^30^ It was estimated that 101 out of every 10,000 cases will develop symptoms after 14 days of quarantine. Documented cases of COVID-19 with incubation periods greater than 14 days have been reported.^31^

Based on the available data, a 14-day quarantine for individuals recently exposed to COVID-19 is reasonable, recognizing that ∼1% of cases will not develop symptoms until after 14 days.^30^ It also could be argued that quarantine should be extended beyond 14 days, particularly for individuals with contacts who are immunocompromised or have other high-risk factors. On the other hand, prolonged quarantine can have adverse consequences by placing a significant burden on working people and social services.^32^

## Duration of Handwashing Should Be at Least 20 Seconds

In addition to social distancing measures, other nonpharmaceutical interventions focus on the importance of personal hygiene. Health organizations universally endorse proper hand hygiene as part of their recommendations for controlling the spread of COVID-19.^6–8^ Handwashing has been demonstrated in multiple studies to effectively remove bacteria from hands and reduce the spread of foodborne and respiratory illneses.^33,34^ The duration of handwashing is an important issue because observational studies indicate that time spent in washing generally falls far short of the recommended duration.^35^ In public restrooms, mean handwashing time is only 5 seconds. Even in hospital settings, handwashing times among health care workers fall short with mean durations of approximately 10 seconds.

The recommended handwashing duration varies among international health organizations (Table 1). However, all organizations recommend a duration of ≥20 seconds. The CDC recognizes that, “determining the optimal length of time for handwashing is difficult because few studies about the health impacts of altering handwashing times have been done.”^36^ A number of studies found that washing hands for 15 to 30 seconds removes significantly more germs than washing for shorter periods.^33,37–39^ Studies of healthy volunteers have shown that 20-second lather times result in a significant 0.5 log reduction in bacterial colony-forming units more than 5-second wash times.^38^ It should be noted that no studies have analyzed the role of handwashing specifically for COVID-19. Nevertheless, the available evidence supports the recommendation of frequent handwashing and a target lather time of at least 20 seconds.

## Conclusion

Reflecting the scant scientific data, the guidelines offered by public health organizations differ from each other in their specific recommendations for personal spacing distance and duration of handwashing. Recent studies contest the presumption that infectious risk is negligible at a 6-foot distance for COVID-19. More research is needed to resolve these areas of uncertainty. Although exact thresholds and relative value of individual measures are unclear, data from population-level, observational, and simulation modeling studies support the importance of social distancing and other nonpharmaceutical interventions in the mitigation of the COVID-19 pandemic. By “flattening the curve,” nonpharmaceutical interventions reduce and delay transmission within the population, prevent medical resources from becoming overtaxed, and buy time for the development of more definitive therapies. As countries have begun to reopen and social distancing measures have become more lax, the resurgence of cases in many regions has been an unwelcome confirmation of the importance of these public health interventions.
